# Heterotopic Ossifications in a Mouse Model of Albright Hereditary Osteodystrophy

**DOI:** 10.1371/journal.pone.0021755

**Published:** 2011-06-29

**Authors:** David L. Huso, Sarah Edie, Michael A. Levine, William Schwindinger, Yingli Wang, Harald Jüppner, Emily L. Germain-Lee

**Affiliations:** 1 Department of Molecular and Comparative Pathobiology, Johns Hopkins University School of Medicine, Baltimore, Maryland, United States of America; 2 Institute of Genetic Medicine, Johns Hopkins University School of Medicine, Baltimore, Maryland, United States of America; 3 Division of Endocrinology and Diabetes, The Children's Hospital of Philadelphia, and Department of Pediatrics, University of Pennsylvania School of Medicine, Philadelphia, Pennsylvania, United States of America; 4 Weis Center for Research, Geisinger Clinic, Danville, Pennsylvania, United States of America; 5 Department of Genetics and Genomic Sciences, Mount Sinai School of Medicine, New York, New York, United States of America; 6 Endocrine Unit, Massachusetts General Hospital, Boston, Massachusetts, United States of America; 7 Pediatric Nephrology Unit, Massachusetts General Hospital, and Harvard Medical School, Boston, Massachusetts, United States of America; 8 Department of Pediatrics, Johns Hopkins University School of Medicine, Baltimore, Maryland, United States of America; 9 Albright Clinic, Kennedy Krieger Institute, Baltimore, Maryland, United States of America; 10 Hugo W. Moser Research Institute at Kennedy Krieger, Baltimore, Maryland, United States of America; University of Western Ontario, Canada

## Abstract

Albright hereditary osteodystrophy (AHO) is characterized by short stature, brachydactyly, and often heterotopic ossifications that are typically subcutaneous. Subcutaneous ossifications (SCO) cause considerable morbidity in AHO with no effective treatment. AHO is caused by heterozygous inactivating mutations in those *GNAS* exons encoding the α-subunit of the stimulatory G protein (Gα_s_). When inherited maternally, these mutations are associated with obesity, cognitive impairment, and resistance to certain hormones that mediate their actions through G protein-coupled receptors, a condition termed pseudohypoparathyroidism type 1a (PHP1a). When inherited paternally, *GNAS* mutations cause only AHO but not hormonal resistance, termed pseudopseudohypoparathyroidism (PPHP). Mice with targeted disruption of exon 1 of *Gnas* (*Gnas* ^E1−/+^) replicate human PHP1a or PPHP phenotypically and hormonally. However, SCO have not yet been reported in *Gnas* ^E1+/−^ mice, at least not those that had been analyzed by us up to 3 months of age. Here we now show that *Gnas* ^E1−/+^ animals develop SCO over time. The ossified lesions increase in number and size and are uniformly detected in adult mice by one year of age. They are located in both the dermis, often in perifollicular areas, and the subcutis. These lesions are particularly prominent in skin prone to injury or pressure. The SCO comprise mature bone with evidence of mineral deposition and bone marrow elements. Superficial localization was confirmed by radiographic and computerized tomographic imaging. *In situ* hybridization of SCO lesions were positive for both osteonectin and osteopontin. Notably, the ossifications were much more extensive in males than females. Because *Gnas* ^E1−/+^ mice develop SCO features that are similar to those observed in AHO patients, these animals provide a model system suitable for investigating pathogenic mechanisms involved in SCO formation and for developing novel therapeutics for heterotopic bone formation. Moreover, these mice provide a model with which to investigate the regulatory mechanisms of bone formation.

## Introduction

Mutations in *GNAS* exons that encode the alpha-subunit of the stimulatory G protein (Gα_s_) lead to Albright Hereditary Osteodystrophy (AHO), which is characterized by brachydactyly, brachymetacarpia, short stature, and frequently subcutaneous ossifications (SCO), i.e. heterotopic bone formation [1]–[4]. In some cases, ossifications are limited to the dermis and subcutaneous tissue whereas in others the ossifications are deeply invasive [2], [4]. It is the only monogenic condition in which *de novo* ossifications form subcutaneously and remain limited to the skin, but both the etiology of the ossifications and their extent of invasiveness are as yet a mystery. When *GNAS* mutations are inherited maternally, AHO is associated with the development of pseudohypoparathyroidism type 1a (PHP1a), i.e. PTH-resistance leading to hypocalcemia and hyperphosphatemia, resistance to several other peptide hormones that mediate their actions through G protein-coupled receptors, as well as obesity and various degrees of cognitive impairment. When the same mutations are inherited paternally, affected individuals develop AHO, including SCO, in the absence of hormonal resistance and obesity, termed pseudopseudohypoparathyroidism (PPHP). Brachydactyly and heterotopic ossifications occur in AHO regardless of the parent of origin of the *GNAS* mutation and most likely reflect the effect of haploinsufficiency in cells that normally express Gα_s_ from both *GNAS* alleles (For review, see [3], [5]–[8]). Dependence on the parental origin of the mutant *GNAS* allele has been found to be secondary to tissue-specific imprinting of Gα_s_ expression based on research conducted in mouse models [9]–[11] as well as human tissues [12]–[19]. In some patients with *GNAS* mutations, SCO can appear without other features of AHO and is therefore termed osteoma cutis, but may later evolve into AHO with or without its associated hormonal abnormalities [20]. Removal of the skin lesions is often followed by a recurrence. The reason for SCO developing only in some patients is not well understood, although SCO tend to form in areas of persistent pressure and worsen with time [3]. Even though loss of G protein-signaling from one parental allele seems to be required for the formation of ossified areas, the underlying mechanisms are incompletely understood.

Interestingly, an entity termed progressive osseous heteroplasia (POH), which occurs predominantly secondary to paternal inheritance of *GNAS* mutations with absence of other AHO features [2], [21], [22], may well represent an extreme variant of AHO involving heterotopic bone formation. The ossifications that occur in POH can be very invasive and may extend below the dermis into the fat and muscle layers [2], [4], thereby leading to skeletal deformities, which can markedly limit a patient's mobility and can be the cause of additional morbidity.

It is unknown why the paternal inheritance of a *GNAS* mutation leads to PPHP in some cases and to POH in others. In addition, it is not known why the ossifications in AHO remain confined to the subcutaneous tissue, yet can be invasive in POH. Studying AHO and POH may thus provide unique opportunities to gain novel insights into the mechanism leading to the formation of ossifications as well as the mechanism that controls the propensity of the ossifications to become invasive.

Targeted, heterozygous disruption of exon 1 of the *Gnas* gene (*Gnas^E1−/+^*) provided a mouse model of AHO, which recapitulated some of the features of the human disease [10], [23], [24]. However, we did not observe heterotopic bone formation in the group of *Gnas^E1−/+^* mice that was examined by palpation or radiographic studies during the first 3 months of life [10]. However, because AHO patients typically show increasing and worsening ossifications with time [3], we therefore evaluated older *Gnas^E1−/+^* mice for the development of SCO and found extensive ossifications [24]. This provided further evidence for the conclusion that our mouse model of AHO replicates the human disease closely, both phenotypically and hormonally [10]. The objective for this study was to further characterize in *Gnas^E1−/+^* mice the ossifications and the timing of their progression to determine whether these animals could provide a system in which to examine the mechanisms leading to heterotopic bone formation. Our ultimate goal would be to utilize this mouse model to find possible methods for the prevention and treatment of heterotopic bone formation, a source of great morbidity in patients with AHO and POH. Additionally, these mice provide a model system with which to investigate the fundamental regulatory mechanisms of bone formation.

## Materials and Methods

### Ethics statement

Tissues were obtained from one human subject with AHO following a protocol approved by the Institutional Review Board (IRB) of the Johns Hopkins Medical Institutions with written informed consent obtained (by E.L.G.-L.). The protocol (#MO10M188) for all mouse studies was approved by the Johns Hopkins University School of Medicine Animal Care and Use Committee and followed federal (NIH) guidelines for the humane and appropriate care of laboratory animals. The animals were housed in cages on ventilated racks in centralized mouse facilities accredited by the American Association for the Accreditation of Laboratory Animal Care.

### Mice

The *Gnas^E1−/+^* mice were generated by targeted disruption of exon 1, as described previously [10], [23]. All mice that carried a mutant maternal allele are hereafter referred to as *Gnas^E1−m/+^* mice and those with a mutant paternal allele as *Gnas^E1+/−p^*. Wild-type mice are referred to as WT. The mice examined in these studies were maintained on a pure 129SvEv background [10] with *ad libitum* feeding of Prolab RMH2500 mouse chow, which contains 0.95% calcium and 0.96% phosphate, and *ad libitum* water.

### Histology

Tissues were obtained from one human subject with AHO and fixed in formalin, demineralized in formic acid solution, embedded in paraffin, sectioned at 5 µm and stained with H&E for histological examination. For mice, the skin was removed intact and was flattened by pinning to index card paper during fixation for 48 hours in cold 4% paraformaldehyde. Skin tissue remained attached to the card and was transferred to 70% ethanol. Skin sections approximately 1–2 mm thick and 2 cm long were cut for embedding, parallel to the direction of the hair from the dorsum, ventrum, and limbs, cutting through any bony foci that were noted. The sections were positioned with the cut surface down and placed into cassettes. In order to section skin on the foot pads, the extremities were disarticulated at the carpus and tarsus joints with the skin in place, fixed in cold 4% paraformaldehyde for 48 hours and decalcified in cold 0.5 M EDTA for 7–10 days. One to two mm thick longitudinal sections from each of the feet were placed into cassettes for embedding. The tissues were embedded in paraffin using routine automatic processing and several 5 µM serial sections made and stained for H&E, Alizarin Red, or von Kossa following routine protocols. Unstained sections were used for non-isotopic *in situ* hybridization. Images were captured using an upright Zeiss Axioskope with a Nikon 1200 DMX digital camera.

### *In situ* hybridization

*In situ* hybridization was performed on sections as described [25], [26] with modifications. Briefly, all slide racks, buckets, and containers were treated with DEPC water (1∶1000) before starting. The mouse osteonectin and osteopontin cDNA fragments were amplified by PCR using specific primers (Osteopontin Forward: 5′ GATGATGACGATGGAGAC-3′; Osteopontin-Reverse: 5′-TGCAAAGTAAGGAACTGTG-3′; Osteonectin Forward: 5′-GGTGCTAACATAGATTTAACTG-3′; Osteonectin Reverse: 5′-AGCCCAATTGCAGTTGAG-3′) and each was cloned into the pCR®II-TOPO® Vector. The plasmids were linearized, and antisense and sense single-stranded RNA probes were generated using T7 and SP6 RNA polymerase with digoxigenin (Roche). Riboprobes (500–600 bp in length) for osteonectin and osteopontin were tested and selected based on the best signal to noise ratio. Sections were deparaffinized, treated with Proteinase K, rinsed, and hybridized with the digoxigenin-labeled riboprobes at 55°C overnight. The next day, slides were washed, blocked, and incubated with HRP-anti-DIG antibody (Dako) for 45 minutes, washed, then incubated with biotinyl-tyramide (Dako) for 8 minutes, washed, and detected with BCIP/Blue (Sigma).

### Radiographic analyses

Three, six, nine, and twelve month old mice were given intraperitoneal injections of 0.2–0.5 mL of 20 mg/mL Avertin [2,2,2,-tribromoethanol (Sigma-Aldrich, St. Louis, MO)] dissolved in tert-amyl alcohol (Sigma-Aldrich)]. Mice were placed in Faxitron (MX20 Specimen Radiography System, Faxitron Corp., Wheeling, IL) and exposed to 20–28 kV for 15 seconds.

### Computerized Tomography (CT) scans

Three month and 12 month old mice were anesthetized with 0.5–1.0% IsoFlo (isoflurane, USP; Abbott Laboratories, North Chicago, IL) delivered via Medical E oxygen at 1 L/min. Mice were scanned using X-SPECT (Gamma Medica-Ideas, Northridge, California) with 512 projections over 10 minutes and with a slice thickness of 0.19 mm. Images were reconstructed using COBRA (Exxim Computing Corporation, Pleasanton, CA). Reconstructed raw images were then analyzed for heterotopic ossifications using Image J software v.1.4.3.67 (1993–2006, Broken Symmetry Soft).

## Results

### Analysis of Human SCO

**Figure 1:**
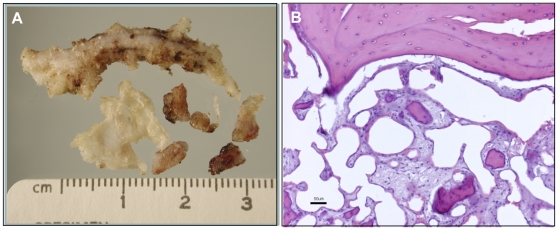
Analysis of human subcutaneous ossification. A) Surgically removed area of ossification from a 20 year old male AHO (−p/+) patient with a documented *GNAS* mutation. The site contained multiple irregular firm masses 0.5–4 cm in length with branching extensions from a central mass. B) Ossified lesion in Figure 1A stained with hematoxylin and eosin. The lesion is composed of mature bone with frequent central bone marrow elements. Scale bar = 50 µm.

Subcutaneous ossifications are a common complication of AHO that can lead to great pain and extensive lesions in patients with this condition. One such site of ossification was surgically resected from the subcutis of the lower leg of a 20 year old male AHO (−p/+) patient with a documented *GNAS* mutation (intron2:c.212+3delAAGT) (informed consent obtained) [27]. The site contained multiple irregular firm masses 0.5–4 cm in length often with branching, tapered extensions from a central mass (Fig. 1A). Histologically the masses were composed of mature bone frequently containing central bone marrow elements (Fig. 1B).

### *Gnas^E1−/+^* mice develop SCO over time

**Figure 2:**
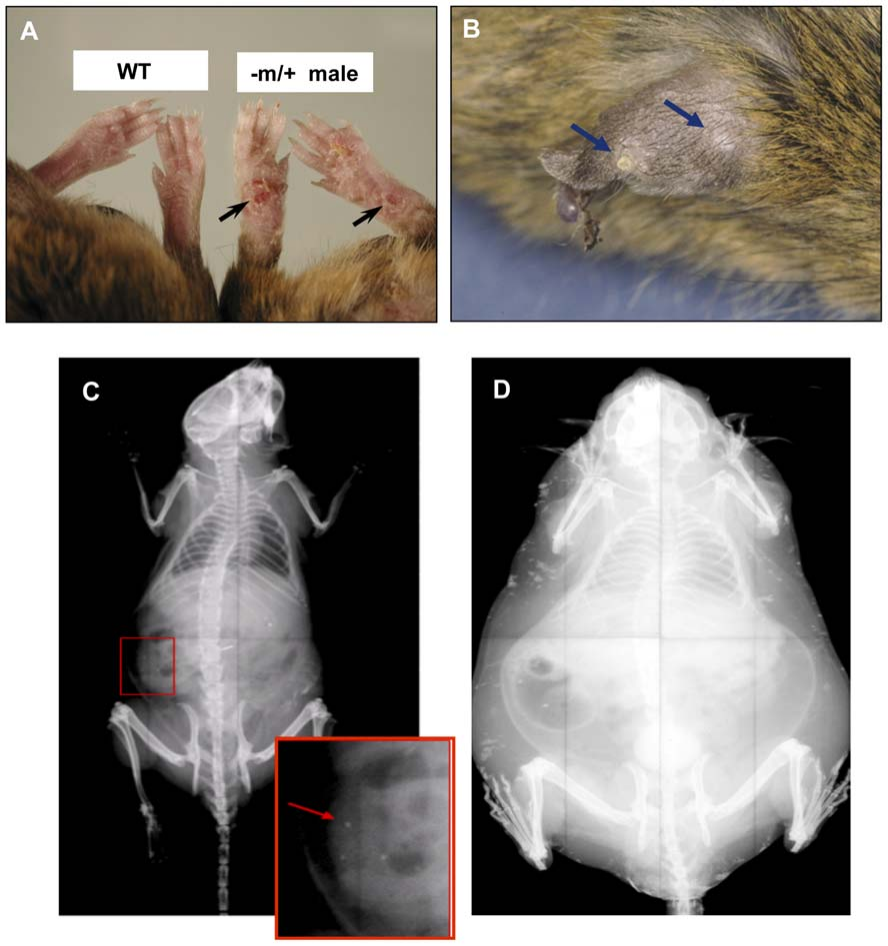
Analyses of subcutaneous ossifications in mice. A) feet of a *Gnas* −m/+ male compared to WT (wild type) male at 12 months of age; B) ear of a *Gnas* −m/+ male at 12 months of age with nodular subcutaneous ossifications; C) X-ray of *Gnas* −m/+ male at 3 months of age showing occasional subcutaneous ossifications; D) X-ray of 12 month −m/+ obese male with extensive subcutaneous ossifications.

Dermal and subcutaneous ossifications were not palpated or visualized in 104 *Gnas^E1−/+^* mice at 3 months of age. We therefore performed physical examinations on 67 older *Gnas^E1−/+^* animals to search for subcutaneous ossifications. Examination of these 12 month old *Gnas^E1−/+^* mice [39 males (21 −m/+, 18 +/−p) and 28 females (15 −m/+, 13 +/−p)] revealed erythematous and mildly erosive footpad lesions in 56 of these mice [38 males (20 −m/+, 18 +/−p) and 18 females (7 −m/+, 11 +/−p)] (Fig. 2A). Palpable subcutaneous nodules were present in 30 mice, all males (15 −m/+ and 15 +/−p). Occasional mice had nodular subcutaneous ear lesions, which were always near the site of ear tags [6 males (4 −m/+, 2 +/−p) and 1 female (−m/+)] (Fig. 2B). Similar lesions were not found in 52 WT mice at the age of 12 months (30 males, 22 females). These lesions are all reminiscent of subcutaneous ossifications that occur in AHO patients based on examination of these lesions in many patients with this disorder (E.L.G.-L., not shown). As in the *Gnas^E1−/+^* mice, the lesions in humans most commonly occur under the skin, especially of the feet, as well as other areas of pressure/trauma. These ossifications were also, occasionally, localized dermally.

Radiographic analyses of 60 mice revealed multiple small densities in the subcutis consistent with mineralized foci that were rare in 3 month old mice and when present, were small with limited radiopacity (Fig. 2C). However, multiple foci were found in all 12 month old mice with targeted disruption of *Gnas* (Fig. 2D). No densities were detected in WT mice. (Details described below.)

**Figure 3:**
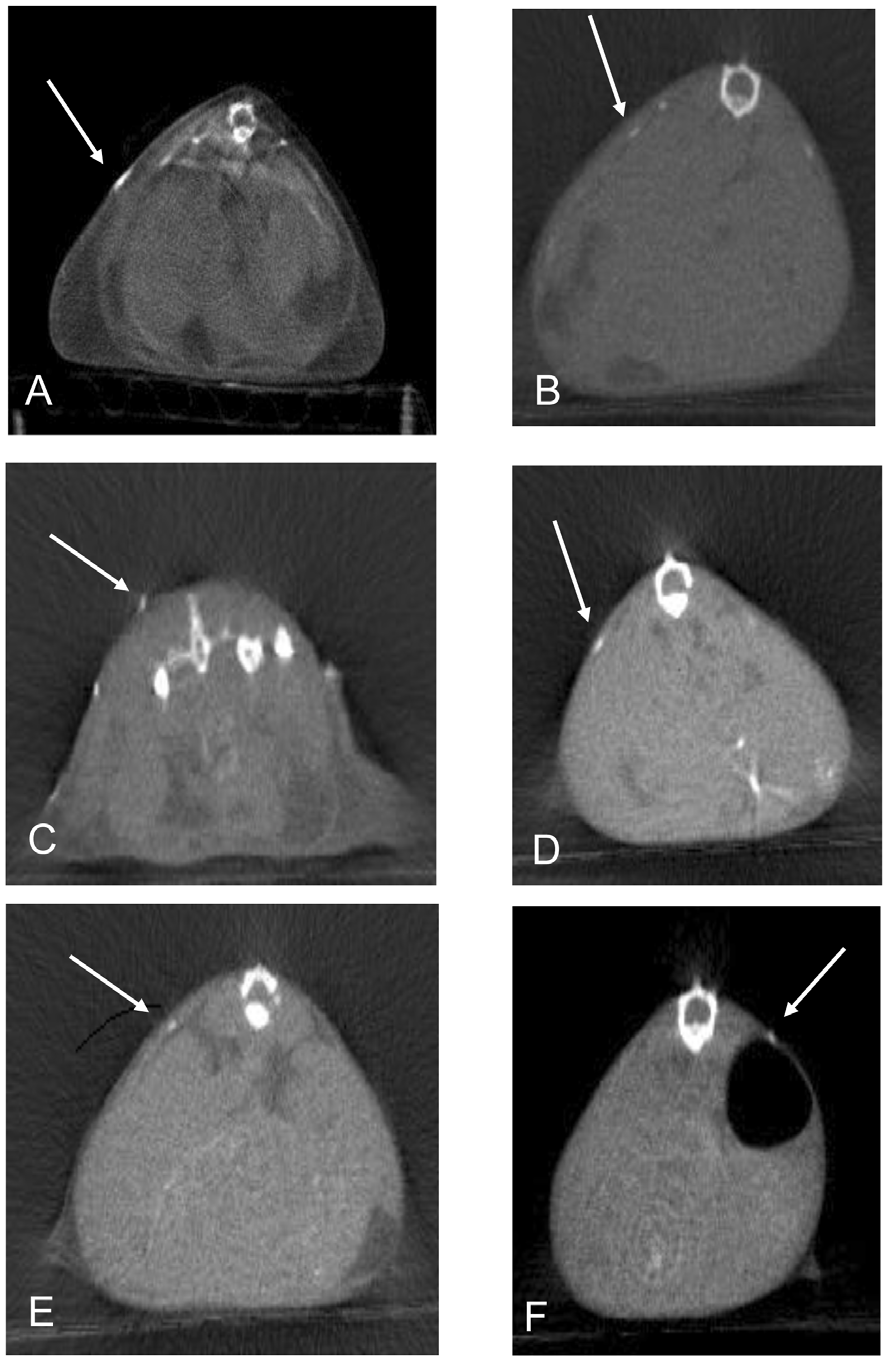
Whole body computerized tomographic (CT) images reveal subcutaneous ossifications. CT scans of 12 month (A–D) and 3 month (E, F) male and female mice with heterozygous targeted disruption of exon 1 in the *Gnas* gene. Arrows indicate a subset of the subcutaneous ossifications. A) male −m/+,. B) female −m/+. C) male +/−p. D) female +/−p. E) male −m/+. F) female −m/+.

Whole body computerized tomographic (CT) images revealed subcutaneous densities that were easily detectable in all 12 month-old *Gnas^E1−/+^* mice examined (male −m/+; female −m/+; male +/−p; female +/−p) (Fig. 3A–D). Consistent with the radiographic studies, no densities by CT were found in WT mice. The CT images confirmed localization of the ossifications to the subcutaneous layer. Only one 3 month old mouse of each gender and genotype (out of four examined for each) had a few very small subcutaneous densities (Fig. 3E, 3F). The X-ray and CT findings thus demonstrated that subcutaneous ossifications occurred with maternally and paternally inherited alleles of both genders, occasionally at 3 months of age and in all mice by 12 months of age; at 12 months of age the ossifications were much more noticeable.

**Figure 4:**
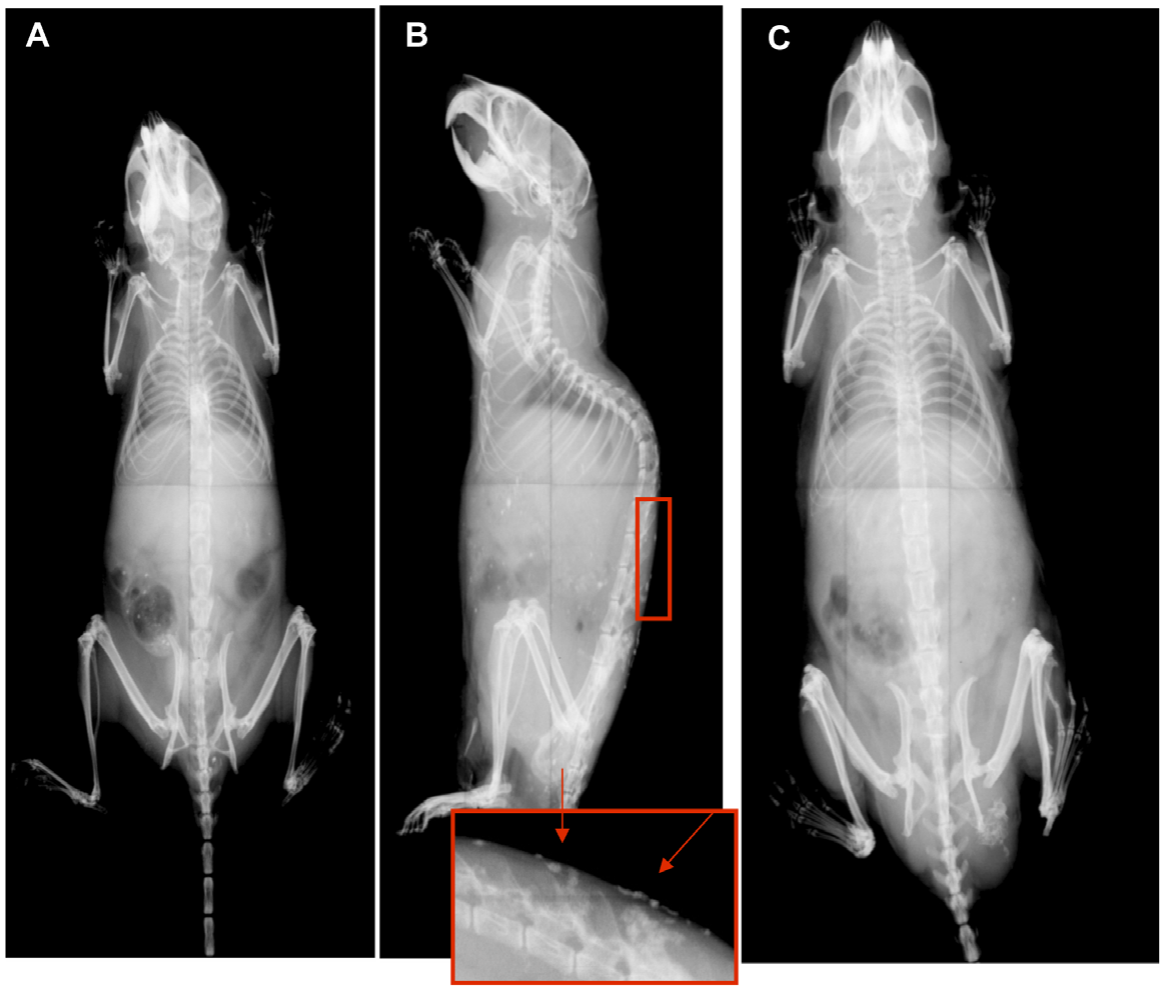
Radiographic analyses of mice reveal multiple subcutaneous ossifications. X-rays of 12 month +/−p and WT mice. A) 12 month +/−p female with no ossifications visualized; B) 12 month +/−p male, inset and arrows demonstrate areas consistent with ossifications; C) 12 month WT without areas of ossifications.

After this observation, the natural history of the evolution of the ossifications was followed in a large number of mice of both genders and both genotypes by serial radiographic analyses (−m/+ and +/−p) at 3, 6, 9, and 12 months (Table 1). With progression of time, more ossifications appeared in both genders. However, the female mice had far less ossifications at all times points examined compared to the males (Fig. 4). In addition, at 12 months the lesions were more severe in males than in females for both maternally and paternally inherited alleles upon histologic analyses (described below). Heterotopic ossifications were not present in any WT mice.

**Table 1:** Radiographic Imaging of Subcutaneous Ossifications Over Time.

Radiographic Imaging of Subcutaneous Ossifications				
	3 Mo	6 Mo	9 Mo	12 Mo
	Number of mice with ossifications/total mice examined (# of foci*)			
Male −m/+	1/13 (1+)	6/13 (2+)	12/12 (3+)	12/12 (4+)
Female −m/+	0/13 (1+)	1/13 (1+)	3/12 (1+)	3/11 (2+)
Male +/−p	1/16 (1+)	8/16 (2+)	15/15 (3+)	15/15 (4+)
Female +/−p	1/18 (1+)	1/17 (1+)	2/15 (1+)	2/14 (2+)

*1+ = 1–3 foci.

2+ = 4–10 foci.

3+ = 11–20 foci.

4+ = >20 foci.

### Histologic analyses of murine SCO

**Figure 5:**
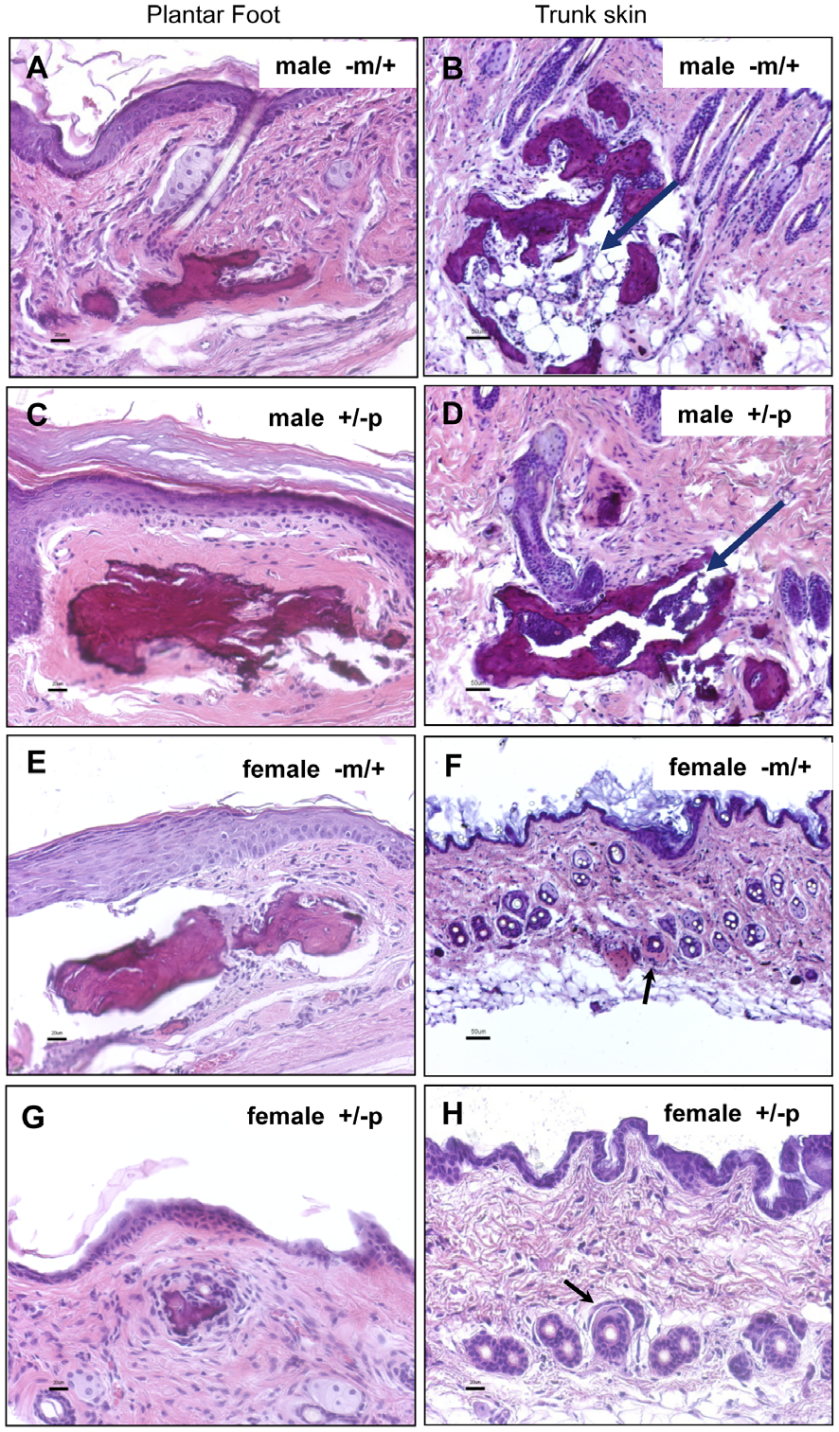
Histological examination of the skin reveals bone formation. Histological examination of the skin from the trunk (Fig. 5B,5D,5F,5H) and feet (Fig. 5A,5C,5E,5G) of male and female −m/+ and −p/+ mice reveal mineralized bone formation often associated with a dense eosinophilic osteoid-like matrix in the dermis and perifollicular areas. No abnormalities were observed in WT mice. Occasional osteoid-like material was present without bone, immediately adjacent to hair follicles, especially in female mice which had milder lesions (Fig. 5F, 5H). Subcutaneous bone frequently contained central bone marrow elements making it histologically indistinguishable from mature skeletal bone (Fig. 5B, 5D). Scale bars = 50 µm.

Histological examination of the skin from the trunk (Fig. 5B, 5D, 5F, 5H) and feet (Fig. 5A, 5C, 5E, 5G) of male and female −m/+ and −p/+ mice revealed mineralized bone formation often associated with a dense eosinophilic osteoid-like matrix in the dermis and perifollicular areas. No abnormalities were present in WT mice. Occasionally a small amount of osteoid-like material was present without bone, immediately adjacent to hair follicles, especially in female mice that had milder lesions (Fig. 5F, 5H). The less frequent and milder lesions in females versus males noted histologically were consistent with radiographic findings (Fig. 4). Subcutaneous bone frequently contained central bone marrow elements making it histologically indistinguishable from mature bone (Fig. 5B, 5D).

**Figure 6:**
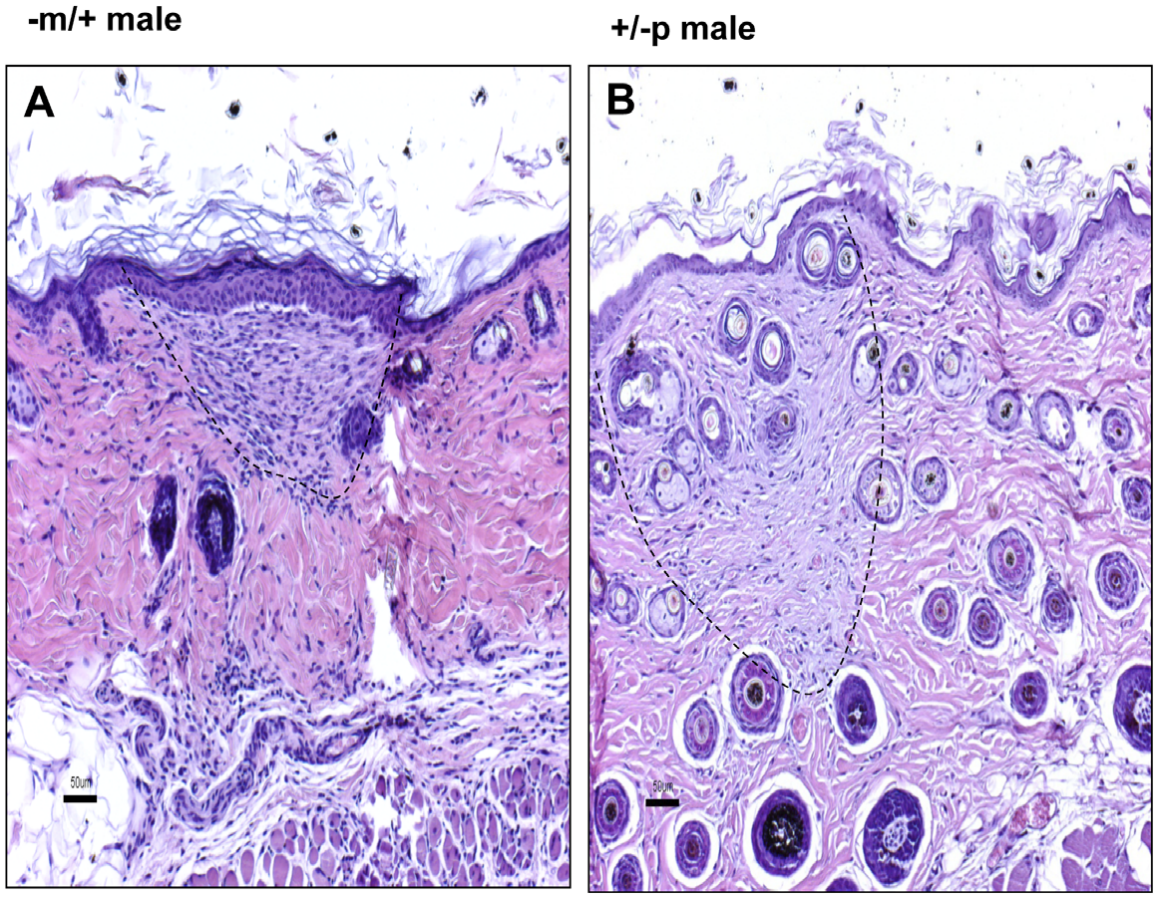
The dermis and subcutis of 3 month old male mice with heterozygous targeted disruption of exon 1 of the *Gnas* gene. The −m/+ and +/−p male and female mice had no heterotopic bone formation. However, there were subtle lesions in the dermis of both −m/+ and +/−p male mice that comprised widely scattered plaque-like areas with pale collagen and increased cellularity in periadnexal areas of the reticular dermis. A) −m/+ male; B) +/−p male; Scale bar = 50 µm.

The dermis of 3 month old −m/+ and +/−p male and female mice had no heterotopic bone formation in the sections of dermis analyzed. However, there were subtle lesions in the dermis of both −m/+ and +/−p male mice (Fig. 6), which contained occasional plaque-like areas in the superficial dermis with increased cellularity and a light amphophilic matrix. The lesions were widely scattered with pale collagen and increased cellularity most pronounced in periadnexal areas of the reticular dermis. These may represent mild or early lesions that precede subcutaneous ossification as these plaque-like areas were not present in sections examined from 3 month old wild type mice.

### Subcutaneous ossifications are mineralized and express osteoblast markers

**Figure 7:**
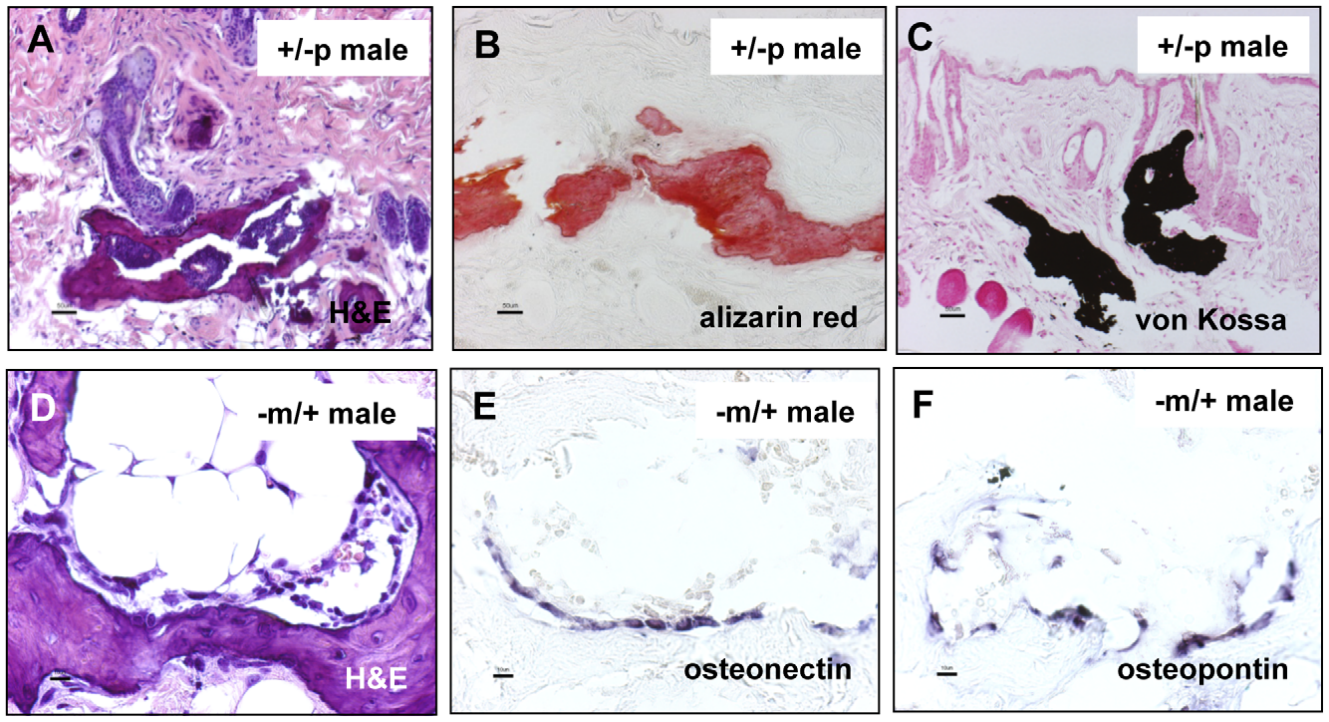
*In situ* hybridization analysis of dermal heterotopic bone using probes to markers of osteogenesis. Nonisotopically labelled osteonectin-specific and osteopontin-specific probes hybridized with cells located in and directly adjacent to foci of dermal heterotopic bone in the mice (no counterstain). Scale bars A, B, C = 50 µm; D, E, F = 10 µm.

**Figure 8:**
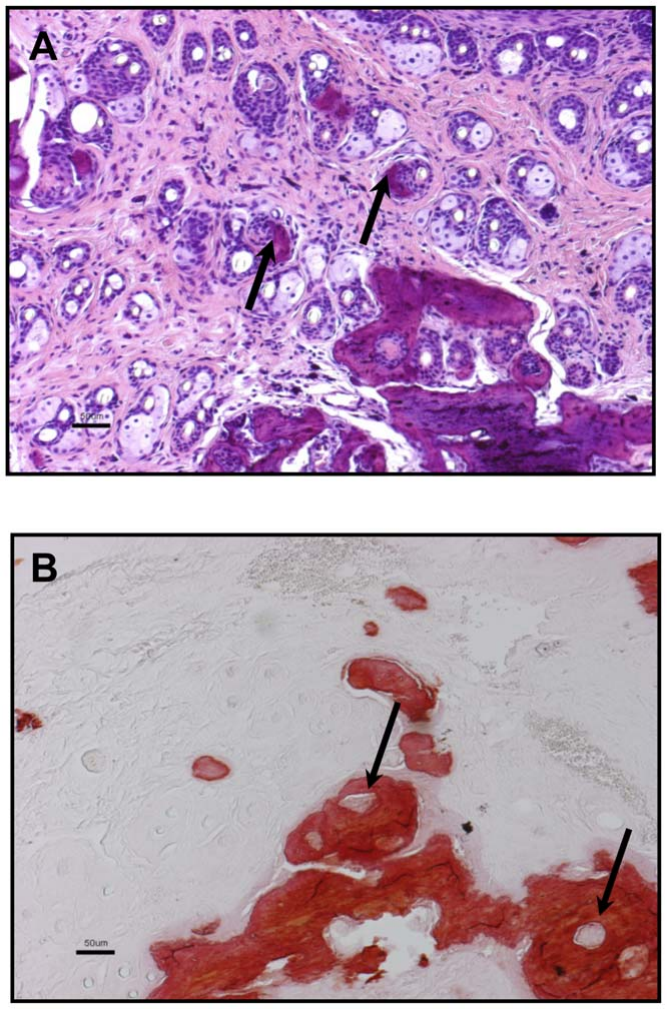
The ossifications appear to originate in the perifollicular areas. Ossifications are emanating near the hair follicle both with hematoxylin and eosin staining (A) as well as Alizarin Red staining (B). Scale bar = 50 µm.

Special stains of subcutaneous bone lesions in 12 month old *Gnas^E1−/+^* mice using Alizarin Red and von Kossa confirmed that the subcutaneous bone osteoid was indeed mineralized (Fig. 7A–C). Sites of subcutaneous ossification (Fig. 7D) also contained cells along the edges of the areas of ossification that expressed osteopontin and osteonectin, consistent with osteoblastic differentiation, as demonstrated by *in situ* hybridization (Fig. 7E, 7F). Upon examination of histologic sections, the SCO appeared to originate in the perifollicular areas, emanating near the hair follicle both with hematoxylin and eosin staining (Fig. 8A) as well as Alizarin Red staining. The Alizarin Red staining encircled the hair follicles in some areas (Fig. 8B). Areas of mineralized bone were often associated with a dense eosinophilic osteoid-like matrix in the dermis and perifollicular areas (Fig. 5B, 5D, 5F, 5H). Occasionally a small amount of osteoid-like material was present without bone, immediately adjacent to hair follicles.

## Discussion

Our mouse model of AHO replicates the human disorder closely, both phenotypically and hormonally [10], and is the first mouse model to exhibit SCO. Although mice with chondrocyte-specific ablation of *Gnas* exhibit ectopic cartilage formation within the metaphyseal region of the tibia [28] and those with targeted heterozygous disruption of exon 2 of *Gnas* (*Gnas^E2 −/+^*) [9] develop subcutaneous fibromas or angiofibromas with associated calcifications [29], there has not been a mouse model that recapitulates the SCO found in AHO in humans until now. We found that our *Gnas^E1−/+^* mice developed extensive and progressive ossifications, thereby differing from the lesions reported in *Gnas^E2 −/+^* mice which had no evidence of bone formation. In our *Gnas^E1−/+^* mice, lesions were very rarely found in the dermis and subcutis of 3 month old mice but were very common in 12 month old mice [3], [10], [24]. Because palpable ossifications in AHO patients frequently increase in number and size with age [3], we investigated this in our *Gnas^E1−/+^* mouse knockout model and found that this was recapitulated. From analyses of mice at 3 months, 6 months, 9 months, and 12 months, it is clear that SCO increase in number and size based on radiographic imaging, although it is possible that the number of SCO are instead constant, with the rapidity of growth differing among the lesions. Computerized tomography verified that these lesions were restricted to the subcutaneous layer and confirmed that the lesions are much less apparent in younger mice. Alizarin Red and von Kossa stains were positive for mineral deposits in the areas surrounding hair follicles at 12 months of age, and many of these areas contained bone marrow elements consistent with true bone formation. This was further confirmed through *in situ* hybridization with probes encoding osteonectin and osteopontin.

Male mice with inheritance of the mutant *Gnas* allele had more severe and widespread heterotopic bone in the subcutaneous tissue than female mice. The lesions in the female mice often resembled precursors of bone consistent with osteoid in the dermis located in the immediate perifollicular areas. The male preponderance raises the possibility that androgens may accelerate the ossification process or estrogen may impair this process. Interestingly, this gender difference has been reported by several human case series in the literature discussing the formation of heterotopic ossifications after surgery [30]–[34]. The predominance in males was reported to be 2–3 times that in females after surgery [35]. Current analyses are underway to investigate whether the male predominance of SCO formation holds true in patients with AHO. Investigation of our mouse model could provide important insight into possible hormonal impacts on the development of heterotopic ossifications and whether hormonal manipulation could aid in prevention, amelioration, and/or treatment of ossification formation.

Although the SCO can occur spontaneously, we have found that in AHO there is a subset of ossifications which occur in areas of pressure and trauma, not only in our patients but also in our mouse model of AHO. Physical examinations of our mice revealed that areas of pressure/trauma such as foot pad lesions and ear tag sites were prone to development of ossifications which occurred more prevalently in males when examined at 12 months of age. This is consistent with the post-operative surgical data in patients as cited previously.

There were no differences in frequency and histological appearance of murine SCO lesions caused by *Gnas* exon 1-disruption of the maternal versus paternal allele. These findings in patients and our genetically manipulated mice thus make it likely that haploinsufficiency, rather than imprinted Gα_s_ expression in an as-of-yet undefined osteogenic precursor, leads to the development of these lesions; these findings are consistent with non-imprinted expression of Gα_s_ in fibroblasts, chondrocytes, and bone cells [28], [36]–[39].

The ossifications appear to originate near the hair follicle. We found extensive areas consistent with heterotopic bone formation located in perifollicular areas in the dermis. There is evidence demonstrating that hair follicle stem cells can differentiate into several cell types including osteogenic lineages [40], thereby raising the possibility that these hair follicle stem cells are progenitors of osteogenesis in the skin. Hair follicle differentiation is tightly controlled by complex pathways involving BMP's and Wnt signaling [41]–[43]. In addition, PTH/PTHrP has been demonstrated to be involved in hair follicle development and the modulation of pathways in both skin and hair follicle cells, thereby implicating a potential role of Gα_s_ in this process [44]–[46].

There is also evidence that mesenchymal stem cells may differentiate into osteogenic lineages. Human mesenchymal stem cells treated with osteogenic medium revealed that knock-down of Gα_s_ with antisense oligonucleotides induced expression of *Cbfa1* and increased osteoblastic determination [47]. Recent data have implicated vascular endothelial cells as multipotent stem-like cells with osteogenic potential, thereby raising the possibility of endothelial cells in the perifollicular areas of the dermis being a possible osteogenic cell precursor [48]. Further studies to determine the cell-type of origin of the heterotopic bone will be crucial in understanding the etiology of the formation of SCO in AHO.

### Conclusion

Our mouse model of AHO replicates the human disorder closely, both phenotypically and hormonally [10], and is the first mouse model to exhibit SCO. Importantly, our findings demonstrate that the *Gnas* exon 1-disrupted mouse model faithfully recapitulates the development of subcutaneous ossifications observed in PHP1a and PPHP patients. In patients with AHO, the ossifications lead to pain and impairment in activities of daily living. There is no effective treatment, and removal of the lesions is often followed by recurrence. Hence, we believe that this mouse model is an ideal system in which to test therapeutic strategies to prevent or limit the growth of ossifications and consequently decrease a source of significant morbidity in patients with AHO and other disorders involving heterotopic bone formation. Additionally, the *Gnas* exon 1-disrupted mouse model represents a valuable tool to elucidate further the molecular and cellular mechanisms involved in ectopic bone formation, as well as the regulatory mechanisms important for bone formation in general.
